# Salivary cortisol concentrations in police officers – a cross-sectional study in Beijing, China

**DOI:** 10.1042/BSR20193406

**Published:** 2020-04-09

**Authors:** Yanxia Zhang, Jie Liu, Yanqing Liu, Wei Lu, Ailian Hou

**Affiliations:** 1 Department of Public Health, Jining Psychiatric Hospital, Jining, China; 2 Drug Rehabilitation Center, Jining Psychiatric Hospital, Jining, China; 3 Department of General Practice, Jining No.1 People’s Hospital, Jining, China; 4 Department of Psychiatry, Huilongguan Hospital, Beijing, China; 5 Department of Neurology, Fuling Central Hospital, Chongqing, China

**Keywords:** Chinese, cortisol, depression, police officers, salivary

## Abstract

Objective: We investigated the relationship between salivary cortisol level and the prevalence of depression 585 police officers working at the Police Departments of Beijing.

Method: Cross-sectional data were obtained from 585 Chinese police officers recruited from Beijing, China. Salivary cortisol was assayed using the chemiluminescence immunoassay. A multiple logistic regression analysis adjusted for potential confounders was used to assess independent associations between salivary cortisol level and depression.

Results: The median age of the included was 38 years (IQR, 29–45), 20.9% were female (*n* = 122). Finally, 15.6% (91/585; 95% CI: 12.6–18.5%) were considered to have depression. The median salivary cortisol level was significantly higher in police with depression than those police without depression [14.5(IQR, 11.9–15.9) nmol/l vs. 11.8(IQR, 9.4–14.2) nmol/l; *P* < 0.001]. The depression distribution across the salivary cortisol quartiles ranged between 5.4% (first quartile) and 26.9% (fourth quartile), *P* for trend <0.001. In multivariate models comparing the second (Q2), third and fourth quartiles against the first quartile of the salivary cortisol, cortisol in Q3 and Q4 were associated with depression, and increased prevalence of depression by 148% (OR: 2.48; 95% CI: 1.55–3.86) and 277% (3.77; 2.12–5.36). Based on ROC curves, the optimal cutoff value of salivary cortisol level to diagnose the depression was 13.8 nmol/l, which yielded the highest sensitivity and specificity [63.8% and 71.7%, respectively; area under the curve (AUC) = 0.695, 95% CI: 0.639–0.751; *P* < 0.0001].

Conclusions: The data showed that elevated levels of salivary cortisol were associated with increased prevalence of depression.

## Introduction

Previous studies have found that routine occupational stress or stressful work conditions have adverse effects on mental health [1]. Police officers are commonly considered to be a high-risk group for the development of mental health disturbances because of the various critical incidents and potential traumatic events they encounter during their career [2]. Previous studies had showed that work stress and trauma exposure may place police at heightened risk for the development of depression symptomatology [3]. Rates of major depression disorder and levels of depression symptoms were found to be higher in police than the general population and those with depression have poorer quality of life [4].

Violanti stated that policing was one of the most stressful jobs in U.S. society [5]. Work disability is related to poor quality of life and those with poorer quality of life are less likely to return to work [6]. Collins and Gibbs [7] pointed out that the proportion of police with measurable mental illness has doubled over the past 10 years. Another study showed that for each standard deviation increase in depressive symptoms, the prevalence ratio (PR) of suicide ideation increased 73 percent in police women (PR = 1.73, 95% CI  =  1.32–2.27) and 67 percent in men (PR = 1.67, 95% CI  =  1.21–2.30) [8]. Furthermore, the mental health problems among police officers can lead to anthropogenic and natural disasters [9]. Therefore, rapidly measurable biomarkers to predict mental health are pivotal for optimized care and allocation of healthcare resources in police.

The major characteristic of the stress response is the activation of the sympathetic nervous system (SNS) and the hypothalamus–pituitary–adrenal (HPA) axis [10]. Any physical or psychological threat to homeostasis triggers release of corticotrophin-releasing hormone in the hypothalamus, and ultimately raises levels of steroid hormones such as cortisol in the blood stream and saliva [11]. Salivary cortisol is frequently used as a biomarker of psychological stress. Psychobiological mechanisms, which trigger the HPA axis can indirectly be assessed by salivary cortisol measures [12]. To date, assessment of cortisol in saliva is a widely accepted and frequently employed method. Due to several advantages over blood cortisol analyses (e.g. stress-free sampling, laboratory independence, lower costs), saliva cortisol assessment can be the method of choice in basic research and clinical environments [13].

The role of cortisol one the glucose, protein and fat metabolism, and cardiovascular reactivity had been proposed [14]. Cortisol hypersecretion is regarded as important in the pathophysiology of major depression, and depressed patients in the community appear to have increased early morning cortisol secretion [15]. Steudte-Schmiedgen et al. [16] found that major depression to be related to long-term attenuation in cortisol secretion. Interestingly, another study suggested that hair cortisol did predict depressive symptoms [17]. Thus, we speculated that level of salivary cortisol was associated with depressive symptoms in Chinese police officers. The aim of this descriptive and cross-sectional study was to investigate the relationship between salivary cortisol level and the prevalence of depression 585 police officers working at the Police Departments of Beijing.

## Participants and methods

### Participants

Between October 2017 and December 2017, Chinese police officers working at the Police Departments of Beijing aged between 25 and 60 years were invited to participate in the study. The exclusion criteria were as following: (1) chronic diseases (e.g. hypertension, diabetes, liver and kidney disease); (2) disabling disease or reduced life expectancy (e.g. severe heart failure, severe respiratory, neurological or psychiatric illness, or late stages of cancer) or difficulty in communication; (3) using of possible or known cognition-impairing drugs in the previous 3-month; (4) inflammatory or infectious disease. Written informed consents were obtained after having provided verbal and written information to participants. Ethics approval was granted by The Ethics Committee for Medical Research at the Fuling Central Hospital. All methods were performed in accordance with the relevant guidelines and regulations.

### Clinical assessment

A validity assessed Self-Administered Questionnaire (SAQ) that was prepared in Chinese, was used to collect details pertaining to sociodemographic and occupational factors from the study participants. Sociodemographic details, including age, sex, race, body mass index (BMI), level of education (undergraduate and postgraduate), current smoker (yes vs. no) and marital status (Unmarried or divorced vs. married), location of residence (urban vs. rural), family yearly income (<200,000 vs. ≥200,000), family problem [little time to spend with families, disharmony of marriage and child-upbringing problems] (yes vs. no), family history of mental illness (yes vs. no), lifestyle factors, self-care and use of medications were documented. Occupational information, including service experience (≤10 years vs. >10 years), infrastructure facilities (satisfactory vs. not satisfactory), welfare facilities (satisfactory vs. not satisfactory), working hours per week (>70 h vs. ≤70 h), consecutive shift work per week (more frequently vs. less frequently), night shifts per month (>6 vs. ≤6) and work satisfaction (satisfactory vs. not satisfactory) were recorded. Basic disease information was obtained from the latest year’s medical report. To ensure the accuracy of the information collected, all demographic data were verified using the information in the household registry cards. Other information was based on self-report

### Physical and depression measurement

Physical exercise was measured using the short version of the International Physical Activity Questionnaire (IPAQ), a self-reporting instrument that asks for an estimate of total weekly physical activity (walking/vigorous and moderate intensity activity) during the previous week. Physical activity levels were categorized into three (low, moderate, and high) categories following the scoring rule of IPAQ [18].

All participants were interviewed using the structured interview. The assessment and diagnostic instrument used in this survey was the Structured Clinical Interview for DSM-IV-TR Axis I Disorders, Research Version (SCID-I/P) [19]. The SCID-I/P is a semi-structured diagnostic screening interview that covers 33 psychiatric disorders described in the fourth edition of the Diagnostic and Statistical Manual (DSM-IV) of the American Psychiatric Association (1994). Currently, it is widely used in diagnostic evaluation, clinical research, and in the training of mental health professionals in China and has been shown to be reliable and valid [20].

The diagnosis of depression was established using SCID through a one-stage screening process. The interviews were conducted by four mental health professionals. The interviewers’ SCID diagnoses were compared with the expert-consensus diagnoses. Inter-rater reliability was examined in 45 participants and the kappa was 0.86. The severity of depressive symptoms was measured with the 17 item Hamilton depression rating scale (HAM-D) [21]. We have used the validated version for the Chinese population.

### Laboratory tests

The salivary samples were collected at 8:00 in the morning. The samples were quickly centrifuged to separate the supernatant from the cells and were then immediately frozen at −80°C until analysis. All analyses were performed in duplicate by the same researcher. Salivary cortisol was assayed using the chemiluminescence immunoassay (Elecsys Cortisol, Roche Diagnostics International AG, Rotkreuz, CH), according to manufacturer instructions. Intra- and inter- assay coefficients of variation were 4% and 6%, respectively. Salivary cortisol levels were expressed in nmol/l. Some of included (*N* = 75) collected salivary samples at 6 time-points: (1) on awakening; (2) at 8:00; (3) at 12:00; (4) at 15:00; (5) at 18:00 and (6) at 21:00. The participants were asked to collect the first (awakening) and the last (bed time) samples in a specific time window: Not later than 10:00 for the first and not later than midnight for the last sample.

### Statistical analysis

The results are expressed as percentages for categorical variables and as median (interquartile range, IQR) for the continuous variables. Correlations among continuous variables were assessed by the spearman rank-correlation coefficient. Proportions were compared by the Chi-square test, and the Mann–Whitney test was used to compare continuous variables between groups.

The relation of salivary cortisol with depression was investigated with the use of logistic regression models. We used crude models and multivariate models and report odds ratios (ORs). ORs were also calculated according to equal quartiles of the distributions of salivary cortisol, and trends across these quartiles were tested by using conditional logistic regression models. For multivariate analysis, we included significant predictors as assessed in univariate analysis. For a more detailed exploration of the salivary cortisol and depression, we also used multivariate analysis models to estimate adjusted OR and 95% CIs of depression for salivary cortisol quartiles (with lowest quartile as reference). The influence of elevated levels of salivary cortisol (defined as more or equal to third quartile) on depression was also performed.

At last, receiver operating characteristic curves (ROC) was used to test the overall prognostic accuracy of the salivary cortisol and results were reported as area under the curve (AUC). All statistical analysis was performed with SPSS for Windows, version 22.0 (SPSS Inc., Chicago, IL, U.S.A.). Statistical significance was defined as *P* < 0.05.

## Results

After excluding 139 participants, 585 Chinese police were included in the study. The response rate of this survey was 80.8% (95% CI: 77.9–83.7%). The reasons for failing to participate included: 52 subjects refused to participate; 62 suffered from chronic diseases; 12 subjects lost samples; and 13 subjects were due to incomplete data. There were no statistically significant differences in sex, age, or location of resident between the participants and non-participants. The median age of the included was 38 years (IQR, 29–45), 20.9% were female (*n* = 122), and median police rank duration was 8.0 (IQR: 4.0–15.0) years. The median HAM-D score was 5 (IQR, 3–10). Finally, 15.6% (91/585; 95% CI: 12.6–18.5%) were considered to have depression. Prevalence of depression was greater among women (35.2%) than men (18.2%). Among those patients with depression, only 6.6% (6/91) start to take psychotropic medications after the diagnosis. The baseline characteristics of those participants were described in Table 1.

**Table 1:** Baseline characteristics according to depressive status

	ALL	Depression	Without depression	*P*^†^
*N*	585	91	494	−
Age (years, IQR)	38 (29–45)	44 (33–49)	39 (27–42)	0.015
Female, *N* (%)	122 (20.9)	32 (35.2)	90 (18.2)	<0.001
Ethnicity-Han, *N* (%)	533 (91.1)	82 (90.1)	451 (91.3)	0.715
BMI (m^2^/kg, IQR)	28.6 (25.4–30.3)	29.6 (26.8–31.7)	28.2 (25.0–29.6)	0.032
Education: Postgraduate or more, *N* (%)	211 (36.1)	35 (38.5)	176 (35.6)	0.605
Marital status: Unmarried or divorced, *N* (%)	133 (22.7)	23 (25.3)	110 (22.3)	0.529
Family problem, *N* (%)	178 (30.4)	36 (39.6)	142 (28.7)	0.039
Location of residence: urban, *N* (%)	422 (72.1)	64 (70.3)	358 (72.5)	0.676
Family yearly income: <200,000, *N* (%)	268 (45.8)	46 (50.5)	222 (44.9)	0.324
Current smoker, *N* (%)	189 (32.3)	33 (36.3)	156 (31.3)	0.380
Family history of mental illness, *N* (%)	65 (11.1)	18 (19.8)	47 (9.5)	0.004
Service experience: ≤10 years, *N* (%)	311 (53.2)	48 (52.7)	263 (53.2)	0.931
Infrastructure facilities: Satisfactory, *N* (%)	388 (66.3)	44 (48.4)	344 (69.6)	<0.001
Welfare facilities: Satisfactory, *N* (%)	331 (56.6)	41 (45.1)	290 (58.7)	0.016
Working hours per week: >70 h, *N* (%)	355 (60.7)	70 (76.9)	285 (57.7)	0.001
Consecutive shift work per week: More frequently, *N* (%)	303 (51.8)	68 (74.7)	235 (47.6)	<0.001
Night shifts per month: >6, *N* (%)	269 (46.0)	55(60.4)	214(43.3)	0.003
Work satisfaction: Not satisfactory, *N* (%)	249 (42.6)	56(61.5)	193(39.1)	<0.001
Physical activity^‡^				0.009
Low	202 (34.5)	43(47.3)	159(32.2)	
Moderate	190 (32.5)	28(30.7)	162(33.8)	
High	193 (33.0)	20(22.0)	173(35.0)	

† Value was assessed using Mann–Whitney *U* test or Chi-Square test.

‡ Physical activity levels were categorized into three (low, moderate, high) categories following the scoring rule of the IPAQ [21].

Data are presented as median (IQR) or number (%); IQR, interquartile range; BMI, body mass index; IPAQ, International Physical Activity Questionnaire

As shown in the Table 1, police with depression were older and more frequently were female, obesity and unmarried or divorced. They were more frequently with family history of mental illness, consecutive shift work per week and night shifts per month. Furthermore, those people were also Less frequently with satisfactory welfare facilities, colleague support and work satisfaction. No association was found between duration of work and the presence of depression.

**Figure 1:**
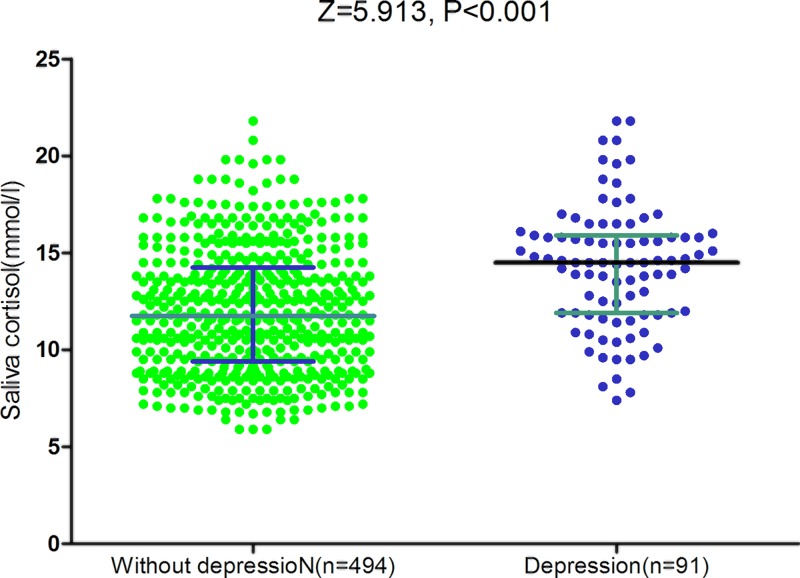
Salivary cortisol level in Chinese police with depression and withoution All data are medians and inter-quartile ranges (IQR); *P* values refer to Mann–Whitney *U* tests for differences between groups.

The data showed that the median salivary cortisol level was 12.1 (IQR, 9.6–14.7) nmol/l. The median salivary cortisol level was significantly higher in police with depression than those police without depression [14.5 (IQR, 11.9–15.9) nmol/l vs. 11.8 (IQR, 9.4–14.2) nmol/l; *P* < 0.001; Figure 1]. The higher salivary cortisol level corresponded to the higher HAM-D score (*r* = 0.615, *P* < 0.001). There was a modest correlation between levels of cortisol and age (*r* = 0.182, *P* = 0.015). There was no correlation between levels of cortisol and sex (*P* = 0.275) and BMI (*P* = 0.095).

The salivary cortisol level was the marker associated with the presence of depression (OR 1.24, 95% CI: 1.15–1.33; *P* < 0.001). Multivariate logistic regression analysis considering traditional risk factors showed a positive relationship between salivary cortisol level and depression when salivary cortisol level was used as a continuous variable (OR, 1.16; 95% CI, 1.07–1.24; *P* < 0.001; Table 2). In addition, sex, age, BMI, family history of psychiatric disorders, infrastructure facilities, working hours per week, consecutive shift work per week, night shifts per month, work satisfaction and physical activity were also associated with the presence of depression unlike other factors (Table 2).

**Table 2:** Multivariate logistic regression for depression in Chinese police (*n* = 585)^†^

Variable	OR (95% CI)	*P*
Age (>40 years vs. ≤40 years)	1.53 (1.22–2.18)	0.036
Sex (Female vs. male)	1.76 (1.28–2.11)	0.003
BMI (>30 vs. ≤30 kg/m^2^)	1.44 (1.19–2.17)	0.041
Family problem (Yes vs. no)	1.33 (0.95–2.04)	0.205
Family history of mental illness (Yes vs. No)	1.68 (1.19–2.46)	0.009
Infrastructure facilities (Satisfactory vs. Not satisfactory)	0.63 (0.42–0.88)	0.003
Welfare facilities (Satisfactory vs. Not satisfactory)	0.84 (0.72–1.03)	0.059
Working hours per week (>70 h vs. ≤70 h)	1.83 (1.27–2.87)	0.011
Consecutive shift work per week (More vs. Less frequently)	2.26 (1.42–3.31)	0.008
Night shifts per month (>6 vs. ≤6)	1.39 (1.08–1.93)	0.027
Work satisfaction (Not satisfactory vs. Satisfactory)	1.51 (1.28–1.88)	<0.001
Physical activity (low vs. moderate + high)	2.42 (1.76–3.14)	<0.001
Salivary cortisol (increase per unit)	1.16 (1.07–1.24)	<0.001

† Adjusted for those significant risk factors that confirmed in the univariate analysis: sex, age, BMI, family history of psychiatric disorders, family problem, infrastructure facilities, welfare facilities, working hours per week, consecutive shift work per week, night shifts per month, work satisfaction, physical activity and salivary cortisol.

widowhood, living with offspring, years of education, and plasma levels of HCY, CRP, IL-6 and HCY.

OR = odds ratio; CI = confidence interval

**Figure 2:**
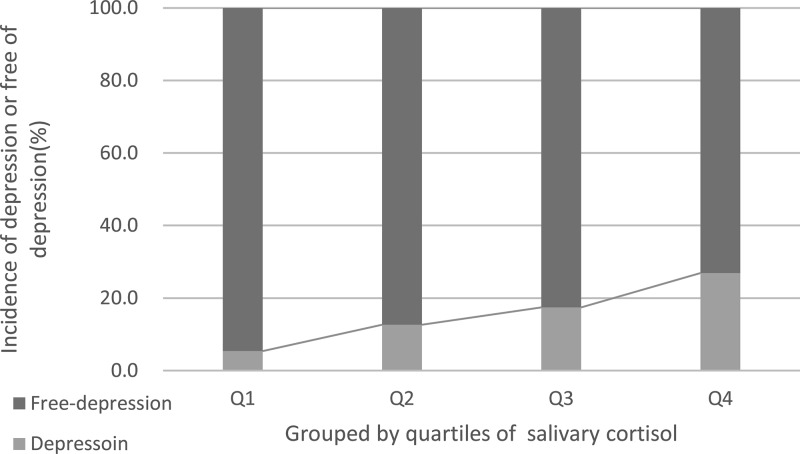
The incidence for depression according to the salivary cortisol quartiles

The probability of depression increased gradually with increasing salivary cortisol quartiles (Figure 2). The depression distribution across the salivary cortisol quartiles ranged between 5.4% (first quartile) to 26.9% (fourth quartile), *P* for trend <0.001. In multivariate models comparing the second (Q2), third and fourth quartiles against the first quartile of the salivary cortisol (Table 3), cortisol in Q3 and Q4 were associated with depression, and increased prevalence of depression by 148% (OR: 2.48; 95% CI: 1.55–3.86) and 277% (3.77; 2.12–5.36). The independent association of salivary cortisol with depression was confirmed using the likelihood ratio test (*P* < 0.001). In a multivariate model using the elevated levels of salivary cortisol (>Q3) versus normal together with the other significant variables, the marker displayed prognostic information (depression: OR for high salivary cortisol, 2.05 [95% CI, 1.38–3.06; *P* = 0.003]).

**Table 3:** Multivariate logistic regression analysis for depression according to salivary cortisol quartiles

Salivary cortisol quartiles^†^	Depression/All	Crude OR (95% CI), *P*	Multivariable-adjusted^‡^, *P*^††^
Q1	8/149	Reference	Reference
Q2	18/142	2.56 (1.08–6.09), 0.029	1.88 (0.93–3.55), 0.076
Q3	26/149	3.73 (1.63–8.53), 0.001	2.48 (1.55–3.86), 0.009
Q4	39/145	6.49 (2.91–14.45), <0.001	3.77 (2.12–5.36), <0.001
Elevated vs. normal	39/145 vs. 52/440	2.75 (1.72–4.38), <0.001	2.05 (1.38–3.06), 0.003

† Salivary cortisol in Quartile 1 (<9.6 nmol/l), Quartile 2 (9.6–12.1 nmol/l), Quartile 3 (12.1–14.7 nmol/l), and Quartile 4 (>14.7 nmol/l). Elevated salivary cortisol level was defined as more than the Q3 level (>14.7 nmol/l).

‡ Adjusted for those significant risk factors that confirmed in the univariate analysis: sex, age, BMI, family history of psychiatric disorders, family problem, infrastructure facilities, welfare facilities, working hours per week, consecutive shift work per week, night shifts per month, work satisfaction, physical activity and salivary cortisol.

†† *P* value for the trend <0.001

Abrreviations: BMI, body mass index; CI, confidence interval; OR, odds ratio.

**Figure 3:**
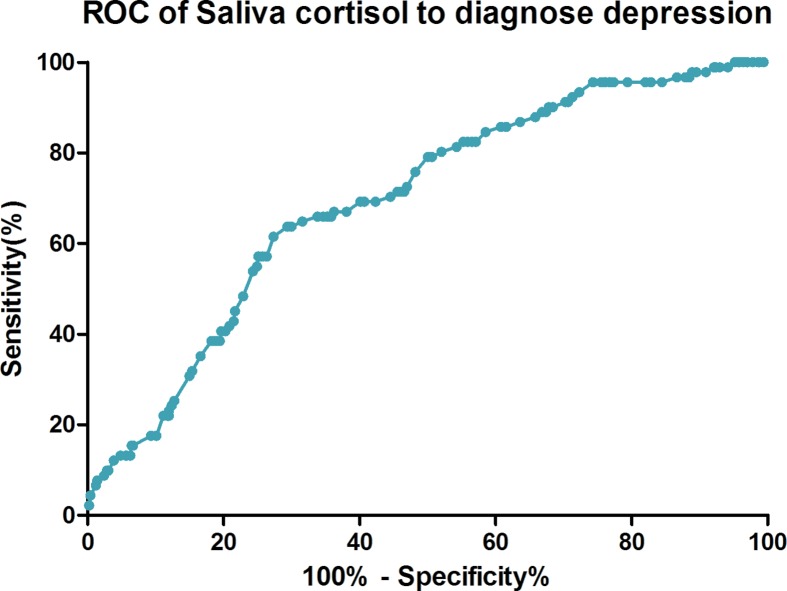
Receiver operator characteristic curve demonstrating sensitivity as a function of 1-specificity for predicting the depression based on the salivary cortisol level

Based on ROC curves, the optimal cutoff value of salivary cortisol level to diagnose the depression was 13.8 nmol/l, which yielded the highest sensitivity and specificity [63.8% and 71.7%, respectively; area under the curve (AUC) = 0.695, 95% CI: 0.639–0.751; *P* < 0.0001; Figure 3]. Further, in our study, we found that an increased prevalence of depression was associated with salivary cortisol ≥13.8 nmol/l (unadjusted OR 4.25, 95% CI: 2.67–6.78). This relationship was confirmed in the dose–response model. In multivariate analysis, there was an increased prevalence of depression associated with salivary cortisol ≥13.8 nmol/l (OR 3.09, 95% CI: 1.64–4.77; *P* < 0.001) after adjusting for above possible confounders.

**Figure 4:**
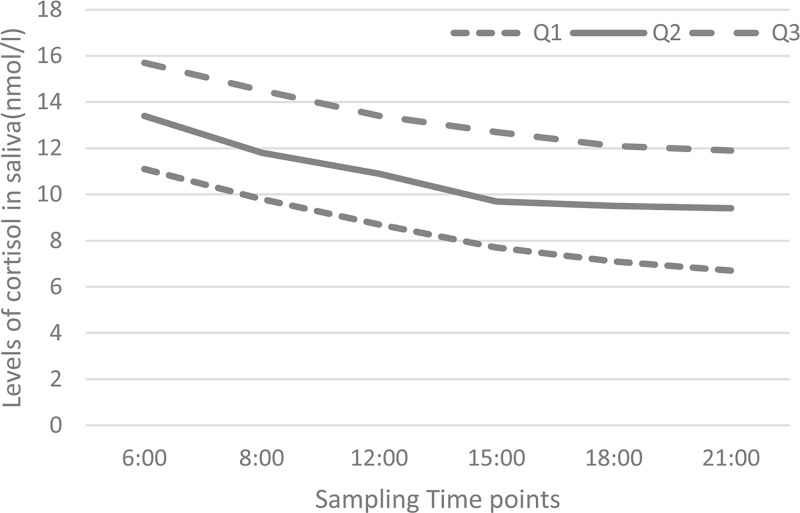
Scatter plots (median, interquartile ranges) of salivary cortisol level in different time-point (*N* = 75)

**Figure 5:**
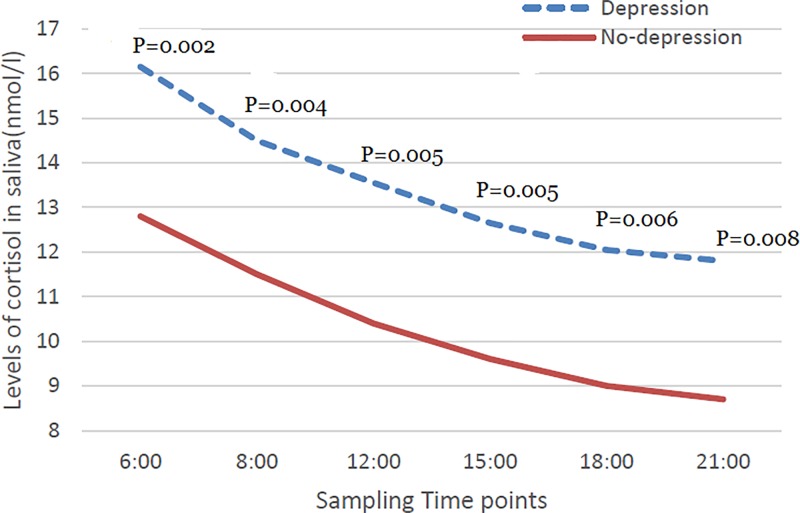
Scatter plots (median) of salivary cortisol level in different time-point in depression group (*N*=12) and non-depression group (*N*=63) *P* values refer to Mann–Whitney *U* tests for differences between groups.

Salivary samples were obtained from six time-point in a subgroup of 75 police, 12 of whom subsequently experienced depression. The result showed the time course of salivary cortisol, showing significant changes with time of sampling (*P* < 0.001), with peak concentrations on awakening (*P* < 0.001, compared with other time-point), falling to a plateau by 15:00 to 21:00 (Figure 4). The Figure 5 also showed that same trend in depression and non-depression groups. Furthermore, the median salivary cortisol level was significantly higher in police with depression than those police without depression in all the six time-point (Figure 5).

## Discussion

To the best of our knowledge, it is the first time to assess the relationship between salivary cortisol level and the prevalence of depression in Chinese police officers. Our main findings were as the following: (1) 15.6% of the police officers were considered to have depression, and prevalence of depression was greater among women (35.2%) than men (18.2%); (2) The median salivary cortisol level was significantly higher in police with depression than those police without depression; (3) Elevated levels of salivary cortisol were associated with increased prevalence of depression and might be useful in identifying police officers at risk for depression for early prevention strategies.

In the present study, we found that the estimated rate of probable depression in police officers was 15.6%. The previous studies had reported that the prevalence of depression has a wide range from 21.6% among Taiwan police officers [4] to 65.6% among Australian police officers [22]. Wickramasinghe et al. [9] found that the estimated prevalence of depression in the police sample was 22.8% (95% CI 19.9–26.1%), and the adjusted prevalence of depression was 10.6% (95% CI 6.6–15.1%). We have a relatively low incidence. This might probably cause by the fact that people with chronic diseases has been ruled out. Furthermore, there were variations in sites of survey, sampling methods, screening instruments, diagnostic criteria, types of interviewer, culture, social and economic status between the various studies [23]. Those variations make it difficult to compare different studies.

Rates of major depression disorder and levels of depression symptoms were reported as higher among police officers than the general population [4]. Our study is also higher than the estimated rate of 2.0% for major depression in metropolitan China [24] and higher than that indicated in another study in four provinces (Shandong, Zhejiang, Qinghai and Gansu provinces) from 2001 to 2005 [25]. They reported the adjusted one-month prevalence of a major depressive episode was 1.55% and 2.60% for men and women, respectively. Another study found that the percent of officers with depression was nearly double (12.0% vs. 6.8%) and officers were nearly four times more likely to sleep less than 6 h in a 24-h period than the general population (33.0% vs. 8.0%) [26].

In the present study, we found that prevalence of depression was greater among women (35.2%) than men (18.2%). Similarly, two previous studies had showed that prevalence of depression was greater among women (22.0%) than men (12.1%) [27], and depressive symptoms were higher among women than men officers (12.5% vs. 6.2%) [8]. Women showed greater HPA axis activation than men and that menopause with loss of estrogens showed the greatest HPA axis dysregulation [28].

Cortisol is an HPA axis-related hormone with a robust circadian rhythm where levels typically peak in the morning hours and decline across the day [29]. In the present study, we also found that salivary cortisol was with peak concentrations on awakening and falling to a plateau at afternoon. In the present study, we found that elevated levels of salivary cortisol were associated with increased prevalence of depression. Police work is considered a stressful occupation that not only involves danger and traumatic event exposure, but also organizational stressors such as lack of administrative support, punishment centered executive philosophies and excessive paperwork [30]. The activity of the HPA axis may be dysregulated in this population lead to a high risk of depression [31,32]. Belanoff et al. [33] found that greater secretion of cortisol might be present in depressed subjects after clinical recovery and withdrawal of medication. A meta-analysis showed that elevated cortisol was associated with depression, with a stronger effect in older inpatients with melancholic or psychotic depression [34]. Furthermore, Grynderup et al. [35] found that a steeper cortisol slope over the day was protective for incident depression, and each 1.0 nmol/l increase in daily mean cortisol concentration was associated with a 47% reduction in depression risk, while each 1.0 nmol/l difference in morning and evening salivary cortisol concentration was associated with a 36% lower risk of depression.

The causal relationship between salivary cortisol and depression needs further study. A previous study demonstrated differential effects of burnout and perceived stress on HPA axis regulation [36]. Some other studies had showed that elevated levels of cortisol might play a role in pathophysiology of depression. One study concluded that in healthy adults elevated free cortisol levels are associated with impaired memory function [37]. Another study suggested that basal cortisol elevation may cause hippocampal damage and impair hippocampus-dependent learning and memory in humans [38]. Chronic exposure to high cortisol levels leads to structural and functional changes in various glucocorticoid receptor-rich brain regions fundamental for emotional and cognitive function, including the hippocampus, amygdala and prefrontal cortex [39]. At last, high cortisol was associated with altered neurotransmitter function, e.g. diminished brain serotonin synthesis, low CSF 5HIAA and increased noradrenergic activity [40]. Further prospective research is warranted.

### Strengths and limitations

Salivary cortisol collection allows for non-invasive timed collection of free cortisol, which is stable for several days before processing, allowing for a valid assessment of the HPA axis in the free‐living state [41]. The assessment of cortisol in saliva has proven a valid and reliable reflection of the respective unbound hormone in blood [13]. Thus, in the present study, we assess the levels of cortisol by the salivary samples. Furthermore, the salivary cortisol was assayed using the chemiluminescence immunoassay with a higher sensitivity and specificity when compared with the enzyme-linked immunosorbent assay.

Some limitations of our study should be taken into account. First, because both salivary cortisol and current depression symptoms were assessed one time at the admission, it is difficult to determine a causal relationship. In addition, the applicability to the workplace may be limited in that salivary cortisol sampling, although non-invasive, may still be impractical or unlikely in the workplace*.* Second, since the response rate of this survey was 80.8%, 19.2% of eligible respondents were not represented in the sample. Third, the present study was lack of functional assessment of HPA axis in parallel and no information about salivary gene expression. Fourth, since the time of awakening is not reported in the manuscript, it is impossible to know whether the samples were collected within the influence of the cortisol awakening response (CAR). This may change drastically the results and the interpretation of the findings. Furthermore, we have to control for the time of awakening in the analyses. Finally, it is possible that the relatively low prevalence of depression symptoms in our sample may be due to a tendency to under-report due to concerns about employment status. In addition, some people with chronic diseases, who more likely suffered from depression has been ruled out. This potential response bias may impact the generalizability of our results.

## Conclusions

The data showed that elevated levels of salivary cortisol were associated with increased prevalence of depression and might be useful in identifying police officers at risk for depression for early prevention strategies. The incidence of depression in police officers were not uncommon, suggesting that steps should be taken to prevent depression and improve the mental well-being of these workers.

## Abbreviations

AUC: area under the curve
BMI: body mass index
DSM: Diagnostic and Statistical Manual
HPA: hypothalamus–pituitary–adrenal
IPAQ: International Physical Activity Questionnaire
IQR: interquartile range
OR: odds ratio
PR: prevalence ratio
ROC: receiver operating characteristic curves
SAQ: self-administered questionnaire
SCID-I/P: Structured Clinical Interview for DSM-IV-TR Axis I Disorders, Research Version
SNS: sympathetic nervous system
